# Autism Spectrum Disorder in a Patient with Bipolar Disorder and Its Relationship with Catatonia Spectrum: A Case Study

**DOI:** 10.3390/brainsci13050704

**Published:** 2023-04-22

**Authors:** Liliana Dell’Osso, Chiara Bonelli, Benedetta Nardi, Giulia Amatori, Ivan Mirko Cremone, Barbara Carpita

**Affiliations:** Department of Clinical and Experimental Medicine, University of Pisa, 56126 Pisa, Italy

**Keywords:** Autism Spectrum Disorder, ASD, high functioning autism, catatonia, bipolar disorder

## Abstract

According to several studies, the prevalence of Autism Spectrum Disorder (ASD) ranges from 2.4 to 9.9 percent among adult mental inpatients. However, subjects with forms of ASD that fit in the high functioning spectrum may remain undiagnosed during childhood and adolescence without reaching clinical attention until they develop in adult life other psychiatric disorders, often characterized by treatment resistance and poor outcomes. The aim of this case report was to evaluate the role of an undiagnosed ASD in the mental illness trajectory and discuss the diagnostic and therapeutic implications. We reported a case of a young man with an undiagnosed ASD that came to clinical attention only after the development of a severe manic episode with mixed and psychotic features and with catatonia in adulthood, despite meeting DSM-5-TR (APA, 2022) diagnostic criteria for ASD since early childhood. This case confirms the need of a timely identification of ASD in order to prevent the development of a mental illness trajectory and to improve the prognosis and the outcome. Moreover, on the heuristic level, this case seems to support the presence of a continuum between ASD and catatonia. In this framework, the use of a questionnaire based on a spectrum model, such as the AdAS Spectrum, could be useful in early diagnosis of ASD without intellectual or language impairment as well as in early detection of subthreshold conditions (broad autism spectrum phenotype or autistic traits), which represents a vulnerability factor for the development of various mental disorders.

## 1. Introduction

Autism Spectrum Disorder (ASD) is a neurodevelopmental disorder with early onset, whose core symptoms are represented by difficulties in communication and social relationships; restricted interests; and repetitive, stereotyped behaviors. ASD has been associated with alterations in brain connectivity with a domino effect on cognitive functions [1]. According to several research, the prevalence of ASD ranges from 0.76% to 2.64% in the general population and from 2.4% to 9.9% among adult psychiatric inpatients [2]. Due to the typical early onset of these conditions, the literature on this topic is mainly focused on child samples, while data about adult manifestations and courses of ASD are growing but still scarce. In this framework, forms of ASD that fit in the high functioning spectrum, especially those without cognitive impairment, may often remain under-recognized until adulthood, when the patient searches for clinical help for the development of other comorbid mental disorders, and, even in this occasion, the underlying ASD may remain misdiagnosed or undetected, with the patient only receiving the diagnosis of the comorbid disorder [3,4,5]. 

While it is of the utmost importance to note that ASD individuals may show average levels of intelligence and no language deficit, the lack of a proper recognition of those forms of ASD in childhood or in adolescence often results in a delay of treatment and assistance, with a detrimental effect on the trajectory of the illness. As reported above, clinical attention may be reached only after the onset of other mental illnesses later in life, thus leading to a greater treatment resistance and worse outcomes, including chronicization and suicidal risk [4,5,6,7,8,9,10].

On the basis of this data, in recent decades, several studies have stressed the importance of paying closer attention to the identification of mild forms of ASD [11,12,13,14,15,16,17,18,19], leading to a progressive increase in the ASD prevalence registered over the past 20 years. Many authors also reported high rates of comorbidity between ASD, including high functioning types, and various psychiatric conditions, ranging from anxiety and mood disorders, with prevalence rates ranging, respectively, between 42–56% and 12–70% [14], to obsessive-compulsive disorder, psychotic disorders, substance abuse, eating and feeding disorders, and personality disorders [6,7,10,19,20,21,22,23]. Another relatively frequent and dramatic complication of high-functioning ASD is catatonia. Catatonia has recently re-gained interest in clinical studies and has been reported to occur in 12–17% of young adults with ASD [24]. Moreover, the majority of autism-related cases of catatonia are identified in people with high-functioning ASD rather than among “classic” autistic patients [25]. In light of the significant percentage of adult ASD patients that goes undetected throughout adolescence due to the lack of an intellectual impairment, it would be appropriate to explore the possibility that they could experience catatonia or catatonic spectrum symptoms at some point in their lives [12,26,27]. Hence, it is clear the necessity for the clinician to sort through the patients’ complicated clinical profiles and be able identify the underlying conditions and the tardive manifestations in order to determine the best course of action.

In light of this framework, the presence of an underlying ASD is not only considered a major vulnerability factor for the development of other psychiatric disorders in comorbidity, but it is also reported to shape, typically in a detrimental way, their presentation, course, and prognosis [10,11,12]. 

In order to assess the broad spectrum of autism symptoms in adults, considering the limits of previous instruments in this field in recent years, the Adult Autism Spectrum Questionnaire (AdAS Spectrum) was developed [13,14,15,16,17,18,19,20,21,22,23,28], which is specifically tailored to assess ASD manifestations in adult subjects with average intelligence and without language impairment, including female phenotypes and subthreshold forms. Since its validation, the AdAS Spectrum has been widely used for research purposes, both in a clinical setting and in non-clinical samples [8,9,10,13,20,21,22,23,29,30,31,32].

In this work, we reported a case of a young man with ASD without intellectual impairment, who, despite manifesting several autistic symptoms since early childhood, including subthreshold catatonic symptoms, came to clinical attention only after the development of a manic episode with mixed features and psychotic features, whose preponderance would have masked the ASD diagnosis if not specifically addressed.

## 2. Case Presentation

X.Y. is a now 26-year-old single man who lives with his parents and two younger sisters. In the medical history of his family, the occurrence of any mental disorder, neurodevelopmental disorder, neurological condition, or substance abuse in first- and second-degree relatives was not reported.

From childhood, he was characterized by both social-phobic and obsessive traits such as introversion, shyness, hypersensitivity to judgment and criticism, and tendency to isolation. He was also described as rigorous, preferring to adhere to a fixed routine and deeply distressed by unexpected events or non-programmed activities. He also showed specific and deep interests, such as physics and astronomy. He preferred to play alone and was not very affectionate, often showing empathy alterations and difficulties in socio-emotional reciprocity. His parents described him as a child who had extremely developed imaginative skills, and he recounted how, from his early childhood, he used to confess his worries and problems to an imaginary self. Since middle and high school, he constantly reported disharmonious scores, excelling in scientific subjects, while showing poor performances in other fields. During adolescence, while maintaining obsessive-compulsive and social-phobic traits, these did not fully meet the diagnostic criteria for a diagnosis of Obsessive Compulsive nor Social-Phobic disorder according to the Diagnostic and Statistical Manual for Mental Disorders (DSM-5). The patient then experienced the progressive onset of frequent mood and energy swings and irritability, in the framework of a cyclothymic–irritable temperament. The onset of psychotic-like symptoms (which the patient identified, during the clinical interview, as the beginning of its mental health problems) was reported to be from the age of 15, when X.Y. developed auditory and visual alterations such as autoscopic hallucinations: in particular, he stated to often saw a sort of doppelgänger of himself, who usually appeared in moments of higher stress and acted as an adviser, giving him opinions about his interests, such as physics and cosmology. These episodes were reported to occur about one or two times a month, when the patient was alone, and were experienced with scarce emotional participation, without worry, and resolved on their own. The patient did not report these experiences to his parents and maintained good functioning in school, family, and social relations. During the summer of the same year, the patient experienced an increasing in restlessness and anxiety due to new social situations that were lived with intense stress, causing elevated rumination; thus, he began to make spontaneous use of cannabis, sporadically at first and then several times a week, experiencing a progressive worsening of the mood swings. However, he did not seek medical attention until the age of 18, when, in August, after the ending of a sentimental relation that lasted 4 months, his mood instability and irritability further worsened, with X.Y. also reporting emotional lability, sadness and loneliness, social anhedonia, asthenia, and hyporexia. Moreover, X.Y showed a growing lack of interest toward school activities that lead to a worsening of academic scores, even in his favorite subjects, a growing reduction in social interaction, and stereotyped and solitary interests, becoming more taciturn and withdrawn from family members. A couple months later, the autoscopic episodes grew in intensity and frequency (multiple times per week), becoming longer and perceived as threatening and terrifying. Those episodes started to show up even in the scholastic environment and become associated with an alteration of the state of consciousness, anterograde amnesia, and automatic behaviors such as writing and walking with closed eyes. The patient also developed a form of suicidal ideation, reporting having been invited by his double to join him in the other dimension. The severity of these symptoms further increased, leading to the interruption of sports and school activities, the complete loss of interest in hedonic activities, and the development of verbal and physical aggressive behaviors if overly stimulated. He began to spend the majority of his days in front of the computer, playing videogames online for up to 10 h a day. For these reasons, X.Y.’s parents finally accompanied him to a first psychiatric consultation in April, which led to his first hospitalization.

Upon entering the ward, the patient presented a flattened, coerced, and poorly reactive affective tone, feelings of emptiness, and disinterest and emotional detachment, with a reduction in basal energy levels. Eye contact was excessively maintained and sometimes fixed. He reported auditory discerptions described as the voice of his thought and disturbing images of a five-legged red and blue horse. During the stay, he underwent an electroencephalography and a cranic magnetic resonance, which did not report alterations of relevant clinical significance. No hallucinatory phenomena occurred during the hospitalization.

At the same time, he was also clinically assessed according to DSM-5 criteria and with the AdAS Spectrum questionnaire, a self-report questionnaire used for the assessment of autistic traits in adult populations, reporting a score of 110/160, greatly above the threshold (of 70) for a potentially clinical diagnosis of ASD (see Table 1). 

In particular, X.Y. reported higher scores in the “Childhood/adolescence” domain and in the “Restricted interests and rumination” domain, reporting, respectively, 85.7% and 80.9% of positive responses. The majority of positive responses in the “Restricted interests and rumination” domain was clinically associated with a strong tendency to ruminative thinking, with a poor ability to process stressful and traumatic events. He also showed high rates of positive responses in the “Verbal communication” domain (77.8%) and on the “Hyper-hypo reactivity to sensory input” domain (76.5%) (where the patient reported amplified perception of visual and auditory stimuli). 

During his hospitalization, X.Y. gradually recovered an affective tone in tune with the outer stimuli, with a recovery of vital momentum and hedonic drive, alongside a congruous and positive planning capacity. He was discharged seven days later with a diagnosis of “Bipolar disorder mixed episode with psychotic features, Substance abuse and Internet Gaming Disorder in subject with Autism Spectrum Disorder”, with a psychopharmacological therapy based on Valproic Acid (up to 1000 mg/die), Paroxetine (20 mg/die), and Aripiprazole (15 mg/die). However, if not considering the ASD diagnosis, he would have possibly met also the criteria for social anxiety disorder and obsessive-compulsive disorder. The ASD diagnosis was according to DSM-5 criteria: despite maintaining an apparent and partial adjustment until adolescence, the patient actually showed persistent deficits in communication and social interaction (deficits in social reciprocity, communicative behaviors, creation and maintenance of relationships), as well as patterns of narrow and repetitive behaviors, interests, or activities. The symptoms were already present in childhood, causing impairment in social and academic functioning. 

In the following period, X.Y. enjoyed a period of substantial psycho-affective well-being characterized by the resumption of interests and activities, good planning abilities, improved social interaction, and disappearance of the autoscopic phenomena; the stereotyped and restricted interests, however, persisted.

In June, X.Y. reported occasional sub-critical episodes of nocturnal pavor, characterized by chest tightness and breathlessness, which, later, grew into spontaneous remission. In September, X.Y. started a university course in Astronomy, for which he had to move to live on his own in another city. Soon, later, he autonomously decided to discontinue the pharmacological therapy, and, subsequently, reported a progressive elevation of mood, increased targeted activity such as football and skateboarding, greater interest and ability in social interactions, and reduction in the need to sleep. In November, the clinical picture evolved with the development of mystical and megalomanic ideas (“God is like a big calculator and I want to do the same: I want to recreate people and everything I have in my head”) that were lived with high emotional involvement, and that led to an worsening social isolation due to the total dedication to behaviors aimed at implementing such ideas and complete occupation of his time in solitary activities on the computer (“I have to become an hacker! I think all the times on how I can become one!”), with consequent impairment of his university career. Soon, later, he experienced a reversal of the sleep–wake rhythm, up to subtotal insomnia, alongside a great reduction in the caloric intake that ultimately led to a second hospitalization lasting eight days, from which he was discharged with a diagnosis of “Bipolar Disorder (manic episode) and Autism Spectrum Disorder” and a psychopharmacological therapy based on Valproic Acid (1500 mg/die), Lithium carbonate (300 mg/die), Aripiprazole (20 mg/die), and Delorazepam (1.5 mg/die); the latter was soon discontinued due to daytime sedation, hypoproxexia, and difficulty in walking with dizziness. During his hospitalization, X.Y. gradually recovered an affective tone in tune with the outer stimuli, with greater thymic stability, restoration of the circadian rhythms, recovery of a planning ability congruous with reality, and a good insight.

In the month of October of the following year, X.Y., after a loss event, experienced a new episode of mood alteration, characterized by a depressed mood with abulia, apathy, anhedonia, reduced energy levels, feelings of guilt and self-depreciation, hypersomnia, and binge drinking, with a progressive social isolation, which led to a decline in social and academic performances. This picture assumed a worsening trend and progressive structuring of suicidal ideation, which led to further hospitalization at our clinic. The hospitalization lasted 24 days, and X.Y. was discharged with a diagnosis of “Bipolar disorder and Autism Spectrum Disorder” and a psychopharmacological therapy based on Valproic Acid (1000 mg/die), Lithium Carbonate (600 mg/die), Aripiprazole (10 mg/die), and Clozapine (62.5 mg/die). At the time of discharge, X.Y. presented a mood oriented towards euthymia, increased willpower and hedonic drive, positive planning, anxious level within the limits, and regularization of the hypnic pattern, with sufficient global psycho-affective compensation, in the absence of self-harming intentions. 

X.Y. maintained a moderate psycho-affective well-being for around 9 months when, following the death of a friend by suicide, he experienced a recurrence of symptoms with feelings of anxiety, dysperceptive phenomena (auditory hallucinations), symptoms of derealization and depersonalization, and ruminativity on the loss. For this reason, it was necessary to increase the clozapine dosage to 150 mg/die, which allowed for an improvement in the symptoms, with a gradual resumption of daily activities and interests. 

A year later, X.Y. decided to enroll to a new university course in Engineering, for which he had to move to a new city. Soon after, he experienced a new recrudescence of the symptoms described above, with accentuation of the ruminativity on the event of loss, ideation of guilt regarding the death of his friend, derealization and depersonalization symptoms, progressive social isolation, hypersomnia, inversion of sleep–wake rhythms, and increased appetite with sporadic binges. He also described auditory misperceptions in the form of the commenting and imperative voice “of the double”, which incited self-aggressive and hetero-aggressive impulses that were not acted upon, the development of the relative ideation of contrasting, intrusive symptoms in the form of dreams related to the traumatic event, to aggressive impulses and the ideation of guilt associated with the development of self-referential and persecutory themes. Two months later, X.Y. showed up without appointment in the clinic, appearing scared and reporting persecutory delusions. He reported that he felt that he had to bring a knife with him when leaving home in order to be able to protect himself due to a sense of threat. He appeared to be partially aware of the delusional nature of his feelings, requesting help for the “fear of being able to use the knife hidden in the backpack”. As a consequence, he was once again admitted in the psychiatric ward and treated with Valproic Acid (1000 mg/die), Lithium salts (124.5 mg/die), Clozapine (150 mg/die), Risperidone (4 mg/die), and Delorazepam (1 mg/die). During his hospitalization, X.Y. gradually recovered an affective tone in tune with the outer stimuli, with a recovery of vital momentum and hedonic drive, alongside a congruous and positive planning capacity. He was discharged fifteen days later with a diagnosis of “Bipolar disorder and Internet Gaming Disorder, past substance abuse disorder in subject with Autism Spectrum Disorder”.

To this day, almost six years later, X.Y. is maintaining good adjustments: although he decided to leave engineering and go back to live with his parents, he is now employed, is able to maintain a stable job, and has a globally adequate social functioning.

## 3. Discussion

We analyzed the case of X.Y., a subject without any intellectual impairment that only came to medical attention after the development of severe psychiatric comorbidities and complications, despite meeting all the diagnostic criteria for ASD since childhood. The lack of detection of the underlying ASD resulted in a lack of timely and adequate treatment, allowing the development of a psychopathological illness trajectory that led to the onset of bipolar disorder in adulthood, with recurrent mixed mood episodes, psychotic features, and suicidal ideation.

Even though X.Y. displayed several behaviors that could be easily referred to the ASD diagnostic dimension during his childhood, due to sufficient social functioning and average academic performance, his symptoms went unnoticed until the appearance, later in life, of other severe psychiatric conditions. This trajectory was in line with recent literature that reported on how individuals with mild or moderate forms of autism, due to the lack of intellectual impairment and, sometimes, the presence of outstanding skills in constrained situations, may remain undiagnosed or be incorrectly classified as having (only) other mental problems [23]. 

Interestingly, during adolescence, due to an increasing anxiety caused by new social situations, X.Y. began a misuse of cannabinoids. This development was in line with the available material regarding the prevalence of substance use disorder (SUD) among individuals with ASD that has acknowledged that high-functioning ASD subjects may be more at risk of undertaking in substance abuse behaviors in order to reduce the anxiety experienced due to their social difficulties [33]. One proposed explanation is that they may develop repetitive “comfort seeking” behaviors due to the sensory hypersensitivity to outside stimuli such as sounds or textures frequently shown by people with ASD, which may include substance abuse and is reinforced by rigid habits [33]. Additionally, the co-occurrence of other disorders frequently associated with ASD, such as generalized anxiety disorder or depression, may make the person even more vulnerable to use drugs or alcohol as a coping mechanism [34,35].

Considering the X.Y. case, if he would have been assessed without acknowledging the presence of his underlying ASD, he would have received multiple diagnoses, such as bipolar disorder in comorbidity with social anxiety disorder and obsessive-compulsive personality disorder. Scientific literature repeatedly suggested that the conventional screening instruments for ASD may have poor discriminant validity when used for adult individuals, especially when high anxiety levels are present [36]. In this context, the AdAS Spectrum questionnaire showed a great ability in discriminating ASD manifestations in adult subjects independently from the presence of other psychiatric manifestations [13,28]. 

Another point of interest is that many other adults with unrecognized ASD are reported to seek clinical attention after minor stressful life events, similar to the presented case, such as the breakdown of a relationship or also after “neutral” life changes such as the beginning of a university course [37]. Subjects with ASD were reported to be highly vulnerable to develop trauma and stress-related symptoms after minor stressors, eventually leading to the presence of a complex post-traumatic stress disorder (cPTSD), which, in turn, may facilitate the development of mood disorders and suicidal ideation [5,6,7,8,9,10,37,38,39,40,41].

Similarly to our case, symptoms belonging to both ASD and psychotic disorders may overlap, leading some mental health professionals to sometimes underrecognize the ASD-related condition.

In fact, the negative symptoms of schizophrenia seem to considerably overlap with those of ASD. For instance, issues with emotional reciprocity, speech delay, or absence in ASD may be explained by blunting of affect or alogia (poor of speech), respectively, in schizophrenia. Both illnesses may also exhibit catatonic symptoms. Since classification systems such as the DSM are based on the observation of specific clinical pictures, differential diagnosis may be challenging in the case of phenotypic similarities. Moreover, in line with the evidence of high rates of comorbidity between ASD and psychosis [42,43], many authors hypothesized the presence of a significant psychotic vulnerability in ASD patients due to their impairments in information processing, which may enhance the risk for a transition to psychosis [43]. Additionally, subthreshold or attenuated symptoms of psychosis comorbid with ASD may further increase the difficulties in correctly distinguishing these diseases [43].

A further peculiar aspect of the case presented is the presence of subthreshold catatonic symptoms scattered throughout the span of X.Y.’s disorders’ course, becoming particularly manifested during adulthood. The current diagnostic criteria for catatonia in the recent DSM-5-TR require three or more of the following symptoms: stupor, waxy flexibility, catalepsy, mutism, posturing, negativism, stereotypes, mannerisms, grimacing, agitation, echopraxia, and echolalia [1]. Interestingly, although X.Y. was not diagnosed with catatonia, he expressed enough symptoms to easily fit in the category at the time of his first hospitalization. Indeed, during his stay, X.Y showed more than three of the twelve symptoms characterizing catatonia-like recurrent episodes of mutism alternated with echolalia; adopted stereotyped behaviors, such as repeatedly walking across the room with closed eyes; and periods of psychomotor agitation characterized by both verbal and physical aggressive behaviors towards his parents and the medical staff. In fact, ASD and catatonia seem to share a variety of clinical traits, including mutism, echolalia, stereotyped movements, repetitive behaviors, negativism, and arousal, which may lead to a delay in the diagnosis of the catatonic symptoms in patients with ASD [25,26]. Moreover, during a routinary examination, X.Y. was administered the recently validated Catatonia Spectrum, a questionnaire that aims to investigate nuclear, subthreshold, atypical, and partial manifestations of the CS during his lifespan [27], on which he obtained a high score (52 out of a maximum of 76) (see Table 2).

This finding supports Lorna Wing’s studies on autism that reported on how the association between ASD and catatonia is not unusual at all [44], with a recent systematic review that reported a catatonia prevalence of 10.4% in people with ASD [24]. Several explanations have been proposed for the clinical overlap between the two conditions, including a common alteration in the GABAergic system [45] or in neural circuits and the size of cerebellar structures [46,47]. A further confirmation comes from animal studies reporting that electroconvulsive therapy, one of the most effective treatments for catatonia, as well as pharmacologically generated seizures, seem to be useful in lowering ASD-like behaviors in mice [48]. Moreover, although of an anecdotal nature, other reports have shown that catatonia may gradually develop over the course of autism, frequently preceded by isolated manifestations and a steady decline in functioning, until it takes on a chronic course, which may further mask the picture and facilitate a diagnosis of schizophrenia spectrum disorders [25,29].

## 4. Conclusions

The use of a spectrum model could help in identifying forms of ASD without intellectual or language impairment as well as subthreshold forms (broad autism spectrum phenotype or autistic traits), which often escape clinical attention in childhood and represent vulnerability factors in the development of various mental disorders. As described above, early detection of high-risk subjects may greatly help to limit the worsening of the clinical picture. In cases similar to the one presented, the clinician’s attention should no longer be focused on how to describe the clinical picture by multiple comorbid diagnoses but rather on the possibility of identifying a single psychopathological trajectory that moves from a neuroatypical substrate such as an unrecognized ASD to a variety of full-blown mental disorders expressing themselves later in life. An early identification of these patients would certainly allow for a better therapeutic framework and a better psychofunctional outcome.

## Figures and Tables

**Table 1:** Score and percentage score reported by X.Y. in the AdAS Questionnaire’s domains.

AdAS Domains	Score	N° of Items	Percentage of Positive Items
Childhood/adolescence	18	21	85.7%
Verbal communications	14	18	77.8%
Nonverbal communications	18	28	64.2%
Empathy	5	12	41.7%
Inflexibility and adherence to routine	25	43	58.1%
Restricted interests and rumination	17	21	80.9%
Hyper-hypo reactivity to sensory input	13	17	76.5%
Total	110	160	68.7%

**Table 2:** Score and percentage score reported by X.Y. in the Catatonia Spectrum Questionnaire’s domains.

Catatonia Spectrum Domains	Score	N° of Items	Percentage of Positive Items
Psychomotor activity	12	16	75%
Verbal response	6	9	66.7%
Repetitive movements	4	7	57.1%
Artificial expressions	4	7	57.1%
Oppositivity	6	7	85.7%
Automatic obedience	3	6	50%
Automatisms	7	10	70%
Impulsivity	10	12	83.3%
Total	52	74	70.3%

## Data Availability

All data generated or analyzed during this study are included in this published article.
